# Elevated urine albumin-to-creatinine ratio increases the risk of new-onset heart failure in patients with type 2 diabetes

**DOI:** 10.1186/s12933-023-01796-6

**Published:** 2023-03-25

**Authors:** Jie Tao, Dasen Sang, Libo Zhen, Xinxin Zhang, Yuejun Li, Guodong Wang, Shuohua Chen, Shouling Wu, Wenjuan Zhang

**Affiliations:** 1 Graduate School of Tianjin Medical University, No. 22, Qixiangtai Road, Heping District, Tianjin, China; 2 Department of Cardiology, Baoding NO.1 Central Hospital, N0.320, Changcheng Street, Baoding, Hebei China; 3 Department of Cardiology, Kailuan General Hospital, 57 Xinhua Road (East), Tangshan, Hebei China; 4 Department of Cardiology, Tianjin Medical University General Hospital, NO. 154, Anshan road, Heping District, Tianjin, China

**Keywords:** Type 2 diabetes, Heart failure, Albuminuria, Urine albumin-to-creatinine ratio, Risk prediction

## Abstract

**Background:**

Although albuminuria has been linked to heart failure in the general population, the relationship between urine albumin-to-creatinine ratio (uACR) and heart failure in type 2 diabetes patients is not well understood. We aimed to investigate the relationship between uACR and new-onset heart failure (HF) in type 2 diabetics.

**Methods:**

We included 9287 Chinese participants with type 2 diabetes (T2D) but no heart failure (HF) who were assessed with uACR between 2014 and 2016. The participants were divided into three groups based on their baseline uACR: normal (< 3 mg/mmol), microalbuminuria (3–30 mg/mmol), and macroalbuminuria (≥ 30 mg/mmol). The relationship between uACR and new-onset HF was studied using Cox proportional hazard models and restricted cubic spline. The area under the receiver operating characteristic curve (AUC), net reclassification improvement (NRI), and integrated discrimination improvement (IDI) were used to see if incorporating uACR into existing models could improve performance.

**Results:**

216 new-onset HF cases (2.33%) were recorded after a median follow-up of 4.05 years. When compared to normal uACR, elevated uACR was associated with a progressively increased risk of new-onset HF, ranging from microalbuminuria (adjusted HR, 2.21; 95% CI 1.59–3.06) to macroalbuminuria (adjusted HR, 6.02; 95% CI 4.11–8.80), and 1 standard deviation (SD) in ln (uACR) (adjusted HR, 1.89; 95% CI 1.68–2.13). The results were consistent across sex, estimated glomerular filtration rate, systolic blood pressure, and glycosylated hemoglobin subgroups. The addition of uACR to established HF risk models improved the HF risk prediction efficacy.

**Conclusions:**

Increasing uACR, even below the normal range, is an independent risk factor for new-onset HF in a type 2 diabetic population. Furthermore, uACR may improve HF risk prediction in community-based T2D patients.

**Supplementary Information:**

The online version contains supplementary material available at 10.1186/s12933-023-01796-6.

## Introduction

Heart failure (HF) is viewed as the chronic, terminal stage of various cardiovascular diseases. The global prevalence of HF is increasing over time because of the aging population and improvement in diagnostic and treatment methods for coronary heart disease (CHD) and valvular heart disease. Epidemiological data have shown that the prevalence of HF is estimated at 1–2% of the general adult population in developed countries and as high as 10% in the population aged > 70 years [1, 2]. The prevalence of HF in Chinese adults was 0.9% and 1.3% in 2003 and 2012–2015, showing an increase of nearly 5 million [3–5] patients when compared with 2003 data. HF patients display a high rehospitalization rate and a similar 5 year survival rate as the patients with malignant tumors, estimated at 50%.

The prevalence of HF in diabetic patients is 2.5–3 times higher than that of the general population [6]. Although diabetic patients have an increased risk of atherosclerosis which might lead to HF through coronary atherosclerosis, the high risk of HF cannot be fully explained by this association. Diabetic microangiopathy is associated with an increased risk of HF and can be assessed by using the urine albumin-to-urine creatinine ratio (uACR). It has been demonstrated that albuminuria was closely related to the occurrence, development, and prognosis of CHD and HF [7–10]. A community-based study also confirmed that the risk of HF was increased by 54–91% with a mild increase in uACR [11].

However, none of the studies mentioned above considered how uACR (spot urine albumin indexed to creatine) affects new-onset HF in type 2 diabetic population. Therefore, this study aimed to analyze the impact of uACR on new-onset HF in patients with T2D and evaluate whether adding uACR to established HF risk models can improve the prediction efficacy of HF risk.

## Methods

### Study cohort

This prospective cohort study comprised in-service and retired Kailuan employees of the Kailuan Group, who participated in the health examination conducted every 2 years in 11 hospitals (Kailuan General Hospital and the affiliated hospitals) from June 2006 to October 2007. The follow-up included an evaluation of HF and death. As urine albumin and creatinine tests were added during the physical examinations in 2014 (5th) and 2016 (6th), diabetic patients who underwent these tests and participated in the 5th and 6th physical examinations were enrolled.

The inclusion criteria were: (1) Patients who participated in the 2014 or 2016 health examination; (2) Participants who met the diagnostic criteria for type 2 diabetes; (3) those who had complete urine albumin and creatinine data, and (4) those patients who agreed for participation and signed informed consent.

The exclusion criteria were: (1) Patients having a history of HF before the physical examination; (2) Patients suffering from valvular and congenital heart diseases.

### Collection of general clinical data and laboratory investigations

All participants completed a questionnaire documenting their sociodemographic status (e.g., age, sex), personal and family health history (e.g., hypertension, diabetes), and lifestyle habits during the on-site visit. Height, weight, and blood pressure measurements, as well as the methods and criteria for determining relevant biochemical parameters, are all described in greater detail elsewhere [12]. Smokers were defined as having smoked at least one cigarette per day on average for the past year, and those who had quit smoking for < 1 year were defined as smokers too. Body mass index (BMI) was calculated as BMI = body weight/height^2^ (kg/m^2^). The estimated glomerular filtration rate (eGFR) was calculated using the Chronic Kidney Disease Epidemiology Collaboration (CKD-EPI) equation [13].

The study was carried out in accordance with the Declaration of Helsinki and was approved by the ethics committee of our hospital. Each participant provided written informed consent.

### Urine albumin and urine creatinine determination and grouping

After an overnight fast, a single random midstream morning urine sample was collected. All participants’ morning urine samples were centrifuged at 600 g for 5 min and stored at − 80 °C until tested. A urine analyzer was used to measure all of the urine samples (N-600, Dirui, Changchun, China). Jaffe’s kinetic method was used to measure urinary creatinine. Turbidimetry was used to measure urinary albumin (DAKO kit, Denmark).

We looked at uACR as a continuous and categorical variable, with normal (uACR < 3 mg/mmol), microalbuminuria (3–30 mg/mmol), and macroalbuminuria (≥ 30 mg/mmol) categories [14, 15].

### Diagnostic criteria

Type 2 diabetes: The American Diabetes Association (ADA) Criteria for Diagnosis of Diabetes (2010) was referred [16].

- 1) History of type 2 diabetes;
- Or 2) Fasting blood glucose (FBG) ≥ 7.0 mmol/L;
- Or 3) Two-hour blood glucose of ≥ 11.1 mmol/L in random plasma glucose test or oral glucose tolerance test;
- Or 4) Hemoglobin A1c (HbA1c) ≥ 6.5% (47.5 mmol/mol).

HF: Chinese Guidelines for the Diagnosis and Treatment of Chronic Heart Failure (2018) was referred [17].

- 1) Symptoms and signs of HF, manifested as shortness of breath, fatigue, palpitations, fluid retention, as well as New York Heart Association (NYHA) heart function grade II and above;
- 2) Modified Simpson’s method: the left ventricular ejection fraction < 50% measured by echocardiography;
- 3) Plasma N-terminal pro-B-type natriuretic peptide ≥ 125 ng/L.

The diagnosis must meet conditions (1) as well as at least one of conditions (2) and (3).

If the time of the first hospitalization for heart failure was earlier than the 5th or 6th physical examination, the patient was considered to have a history of heart failure.

### Follow-up and endpoint events

After the completion of the 5th or 6th health examination, that is, the starting point of follow-up, trained medical staff reviewed the inpatient diagnosis and recorded the end-point events of the participants in the Affiliated Hospitals of Kailuan Group and the Designated Hospitals for Medical and Health Insurance of China every year. The end-point events ware defined as HF during the follow-up. The time of the first event was considered as the end-point for those with > 2 events, and the final follow-up date for those without HF was December 31, 2020. All diagnoses were confirmed by professional physicians according to the inpatient medical records.

### Statistical analysis

Normally distributed measurement data were expressed as mean + sd. Multiple pairwise-comparison between different groups was conducted using a one-way analysis of variance. The least significant difference (LSD) test and Dunnett’s T3 test were used for evaluating the homogeneity of variance and heterogeneity of variance, respectively. Non-normally distributed data were presented as median and centiles (25th and 75th), while the comparison between the groups was performed using the Kruskal–Wallis rank sum test. Enumeration data were presented as frequency and percentage (n, %), and comparisons between groups were performed by the chi-square test. The Kaplan–Meier method was used to calculate the incidence of HF events in each group and the overall population, and a log-rank test was adopted to compare the difference in the incidence of HF.

The uACR was assessed as a categorical and continuous variable. Given a non-normal distribution, uACR was ln-transformed for the continuous model. The effect of different uACR groups and each 1-standard deviation (SD) increase in ln (uACR) on new-onset HF was studied using a multivariate Cox stepwise regression model. Model 1 unadjusted. Model 2 was adjusted for age and gender. Model 3 was further adjusted for SBP, BMI, total cholesterol, HbA1c, eGFR, hemoglobin, smoking, anti-diabetic treatment, antihypertensive treatment, CHD, and atrial fibrillation.

In addition, based on Model 2 (age, gender), Model 4 (WATCH-DM risk score: age, BMI, SBP, DBP, FPG, serum creatinine, HDL cholesterol, CHD) and Model 5 (Williams et al. study model: age, SBP, CHD, Atrial fibrillation, HbA1c, Albumin, BUN, eGFR, smoking), the receiver operating characteristic (ROC) area under the curve (AUC), net reclassification index (NRI), and integrated discrimination improvement (IDI) were used to assess the ability of uACR to improve HF prediction models, respectively.

A spline function curve was plotted to see if there was a linear correlation between uACR and new-onset HF. The multivariable adjusted model include age, gender, SBP, BMI, total cholesterol, HbA1c, eGFR, hemoglobin, smoking, anti-diabetic treatment, antihypertensive treatment, CHD, and atrial fibrillation.

Considering the impact of death on HF during follow-up, a competing risk model for mortality was constructed for the overall population. Furthermore, in order to avoid the influence of CHD, hypertension, and antihypertensive drugs on HF, sensitivity analysis was performed after excluding the above population.

SAS version 9.4 was used for the analysis (SAS Institute, Cary, NC, USA). All statistical analyses were double-tailed, with statistical significance set at *P* < 0.05.

## Results

### Study cohort

**Fig. 1:**
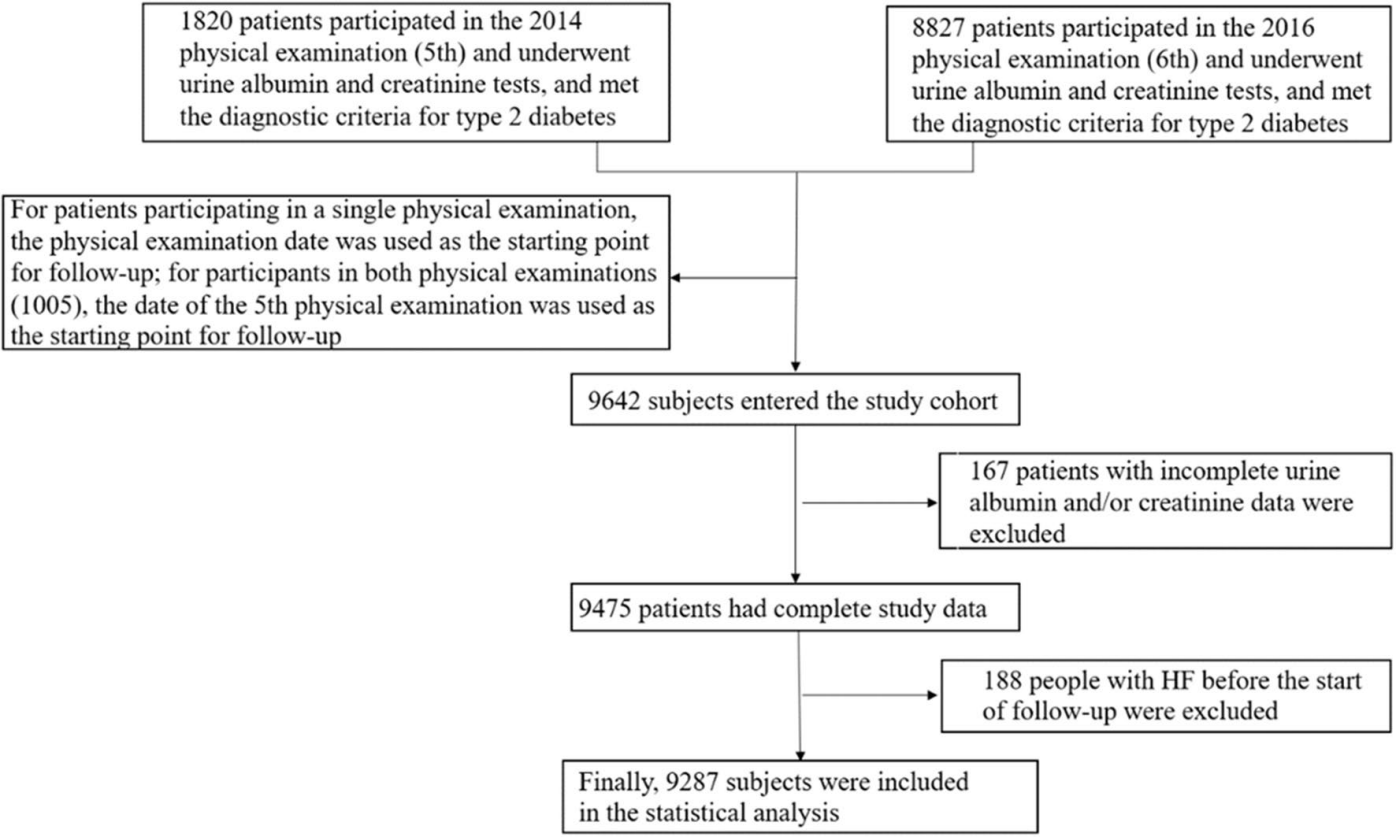
Flowchart of the current study

A total of 1820 T2D patients participated in the 5th physical examination, which included urine albumin and creatinine tests; 8827 patients participated in the 6th physical examination, which included urine albumin and creatinine tests. However, 9642 patients were included in the study after excluding 167 and 188 patients who had incomplete urine albumin and creatinine data and a history of HF before the physical examination, respectively. Subsequently, 9287 patients with T2D were finally included in the statistical analysis (Fig. 1).

### Baseline characteristics

The observed patients’ baseline age was 61.10 ± 9.97 years and included 6815 (73.37%) males and 2472 females (26.63%). The systolic blood pressure (SBP) was 146.70 ± 20.69 mmHg, HbA1c was 7.60 ± 1.65% (57.36 ± 18.21 mmol/mol), and uACR was 1.67 (0.80, 4.61) mg/mmol; 65.9% of the overall population had uACR in the normal range (n = 6120), and 28.1% and 6.0% of them had microalbuminuria and macroalbuminuria, respectively. When compared with the normal uACR, the patients with microalbuminuria and macroalbuminuria exhibited higher SBP, total cholesterol, low-density lipoprotein cholesterol (LDL-C), triglycerides, HbA1c, BMI, high sensitivity C-reactive protein (hs-CRP), heart rate, hypertension prevalence, and HF prevalence as well as a lower eGFR level (Table 1).

**Table 1:** Baseline characteristics overall and by uACR categories in participants

	Overall 9287	< 3 mg/mmol 6120	3–30 mg/mmol 2611	≥ 30 mg/mmol 556	*P-*value
Heart failure	219 (2.33%)	72 (1.18)	84 (3.22)	60 (10.79)	< 0.001
Male, n(%)	6815 (72.37)	4452 (72.75)	1887 (72.27)	415 (74.64)	0.513
Age, year	61.10 ± 0.97	60.42 ± 9.78	62.17 ± 10.05	63.57 ± 10.09	< 0.001
uACR^a^, mg/mmol	1.67 (0.80 4.61)	1.01 (0.63–1.64)	6.18 (4.13–11.29)	61.66 (42.68–114.64)	< 0.001
SBP, mmHg	146.70 ± 20.69	143.56 ± 19.72	151.82 ± 20.74	157.01 ± 22.30	< 0.001
DBP, mmHg	82.92 ± 11.03	81.95 ± 10.52	84.57 ± 11.64	86.01 ± 12.04	< 0.001
Heart rate, beats/min	77.48 ± 12.82	76.58 ± 12.43	78.99 ± 13.22	80.39 ± 13.58	< 0.001
Waist circumference, cm	90.72 ± 9.62	90.32 ± 9.57	91.38 ± 9.69	91.96 ± 9.63	< 0.001
BMI, kg/m^2^	25.81 ± 3.43	25.58 ± 3.32	26.14 ± 3.58	26.54 ± 3.64	< 0.001
Triglycerides^a^, mmol/L	1.53 (1.05–2.30)	1.45 (1.00–2.18)	1.68 (1.15–2.60)	1.85 (1.26–2.83)	0.008
Total cholesterol, mmol/L	5.48 ± 1.17	5.42 ± 1.13	5.58 ± 1.21	5.81 ± 1.38	< 0.001
HDL cholesterol^a^, mmol/L	1.37 (1.18–1.62)	1.39 (1.19–1.64)	1.34 (1.16–1.58)	1.32 (1.12–1.57)	0.505
LDL cholesterol, mmol/L	3.24 ± 0.95	3.20 ± 0.92	3.30 ± 0.99	3.42 ± 1.05	0.001
FBG, mmol/L	9.10 ± 3.26	8.54 ± 2.95	10.04 ± 3.48	10.77 ± 3.86	< 0.001
HbA_1c_, %(mmol/mol)	7.60 ± 1.65, 57.36 ± 18.21	7.32 ± 1.54, 56.50 ± 16.79	8.10 ± 1.74, 65.05 ± 19.40	8.40 ± 1.84, 68.31 ± 19.95	< 0.001
Hemoglobin, g/L	150.65 ± 14.51	150.66 ± 14.16	151.00 ± 14.61	148.89 ± 17.47	0.019
Albumin, g/L	44.4 ± 9.07	44.56 ± 8.73	44.33 ± 9.77	43.15 ± 9.21	0.002
BUN, mmol/L	6.08 ± 2.15	5.99 ± 2.06	6.08 ± 2.03	7.05 ± 3.23	< 0.001
Hs-CRP^a^,mg/L	1.10 (0.34–2.76)	0.93 (0.29–2.40)	1.41 (0.47–3.39)	1.68 (0.68–3.65)	0.264
eGFR, mL/min/1.73 m^2^	90.95 ± 16.92	91.93 ± 16.09	90.30 ± 16.70	83.29 ± 23.25	< 0.001
smoking	3123 (33.63)	2092 (34.18)	860 (32.94)	171 (30.76)	0.177
Hypertension, n (%)	5272 (56.77)	3161 (51.65)	1719 (65.84)	392 (70.50)	< 0.001
Atrial fibrillation, n (%)	100 (1.09)	55 (0.90)	38 (1.46)	7 (1.28)	0.063
CHD, n (%)	493 (5.31)	309 (5.05)	146 (5.59)	38 (6.83)	0.148
Anti-diabetic treatment, n (%)	3947 (41.91)	2294 (37.46)	1269 (48.60)	338 (60.79)	< 0.001
Insulin, n (%)	1678 (17.82)	906 (14.80)	555 (21.26)	191 (34.35)	< 0.001
Oral medicine, n (%)	2274 (24.15)	1391 (22.73)	715(27.38)	148(26.62)	< 0.001
Antihypertensive treatment, n (%)	3899 (41.98)	2461 (40.21)	1136 (43.51)	302 (54.32)	< 0.001
ACEI or ARB, n (%)	923 (9.94)	501 (8.19)	311 (11.91)	111 (19.96)	< 0.001
Beta-blocker, n (%)	471 (5.07)	267 (4.36)	155 (5.94)	49 (8.81)	0.710
Calcium channel blocker, n (%)	869(9.36)	434 (7.09)	309 (11.83)	126 (22.66)	< 0.001
Diuretic, n (%)	280 (3.01)	121 (1.98)	104 (3.98)	55 (9.89)	0.001
Others, n (%)	2460 (26.49)	1682 (27.48)	643(24.63)	135 (24.28)	0.001

^a^Expressed in M (Q1–Q3)

### Cumulative incidence of HF events in each uACR group

Following a median follow-up time of 4.05 (3.55, 4 0.48) years, 216 patients (2.33%) developed HF, and 496 patients (5.3%) died of all-cause mortality, respectively.

**Fig. 2:**
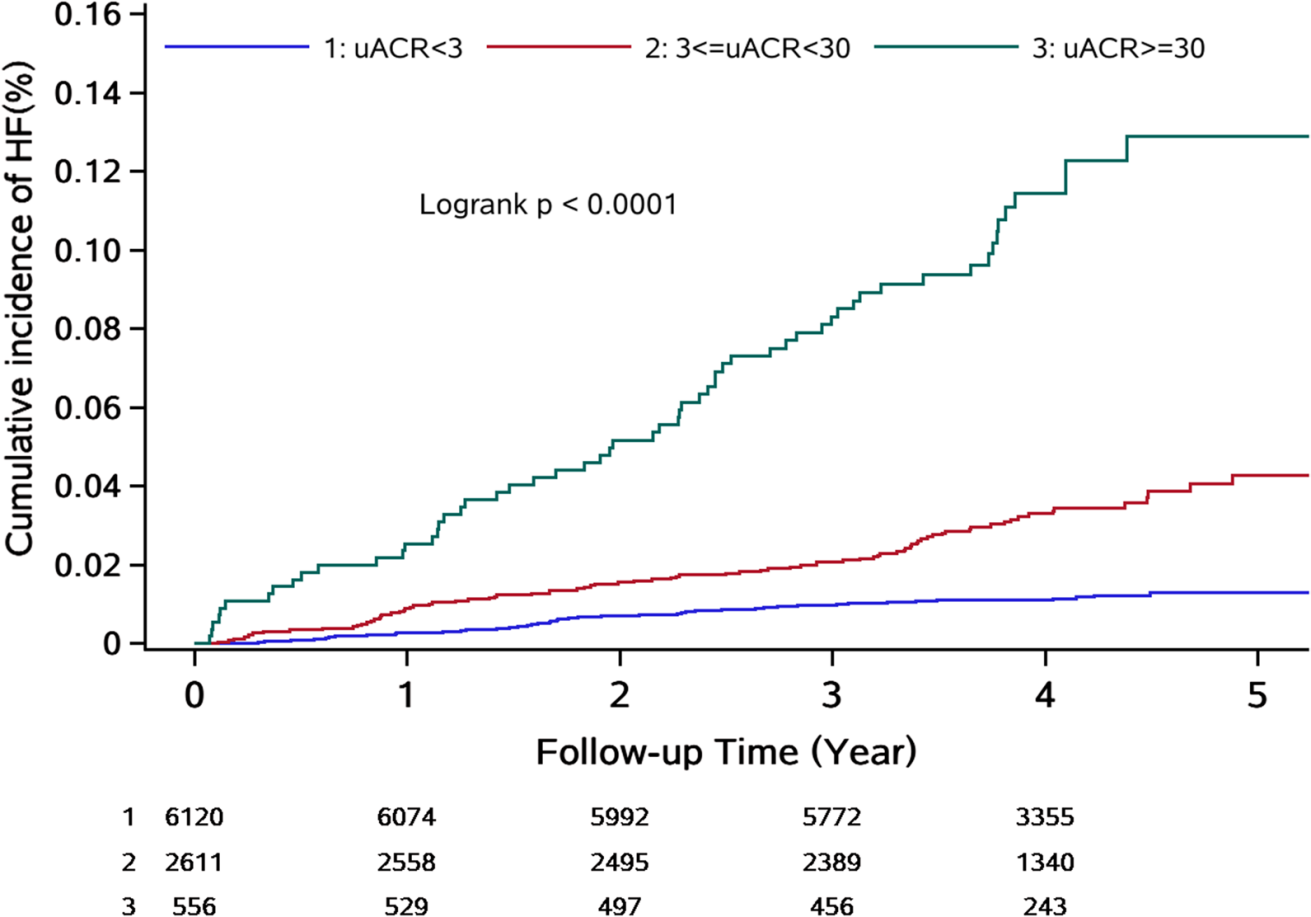
Incidence of heart failure by albuminuria category: albuminuria categories were based on urinary albumin-creatinine ratios (uACR) as macroalbuminuria (uACR ≥ 30 mg/mmol), microalbuminuria (uACR < 30 to ≥ 3 mg/mmol), and normal (uACR < 3 mg/mmol). P < 0.0001 for differences among curves using the log-rank test

The cumulative incidence of HF in all three groups was 1.55%, 3.37%, and 15.07%, respectively. A log-rank test showed a significant difference in the cumulative incidence between the three groups (Fig. 2).

### Multivariate Cox regression analysis of the relationship between uACR and new-onset HF

With the presence or absence of HF as the dependent variable and uACR groups or per 1-SD increase in ln (uACR) as the independent variable, and after adjustment for covariates, the risk of new-onset HF was 2.21 fold (95% CI 1.59–3.06) and 6.02 fold (95% CI 4.11–8.80) higher in the patients with microalbuminuria and macroalbuminuria than in the patients with normal uACR, respectively; the risk of new-onset HF increased by 89% (95% CI 68–113%) per 1-SD increase in ln (uACR) (Table 2).

**Table 2:** Hazard ratios (HR) and 95% confidence intervals of uACR for heart failure

uACR category	No	Median follow-up (years)	Incident heart failure (%)	Incidence rate (/1000 person-years)	Model1	Model 2	Model 3
< 3 mg/mmol	6120	4.04	72 (1.18)	2.89	1	1	1
3–30 mg/mmol	2611	4.06	84 (3.22)	8.10	2.81 (2.05, 3.84)	2.48 (1.81, 3.41)	2.21 (1.59, 3.06)
≥ 30 mg/mmol	556	4.08	60 (10.79)	29.29	10.13 (7.19, 14.27)	8.39 (5.94, 11.86)	6.02 (4.11, 8.80)
ln(uACR), Per 1 SD	9287	4.05	216 (2.13)	5.79	2.15 (1.94, 2.37)	2.06 (1.85, 2.28)	1.89 (1.68, 2.13)

Model 1: unadjusted; Model 2: adjusted for age and sex; Model 3: adjusted for age, sex, SBP, BMI, total cholesterol, HbA1c, eGFR, hemoglobin, smoking, anti-diabetic treatment, antihypertensive treatment, CHD and atrial fibrillation

Additionally, we constructed a competing risk model for mortality for the overall population to eliminate the impact of all-cause mortality events on the outcome during follow-up and obtained consistent results (Additional file 1: Table S1).

### Restrictive cubic spline Cox proportional hazards model was used to analyze the relationship between uACR and the risk of new-onset HF

**Fig. 3:**
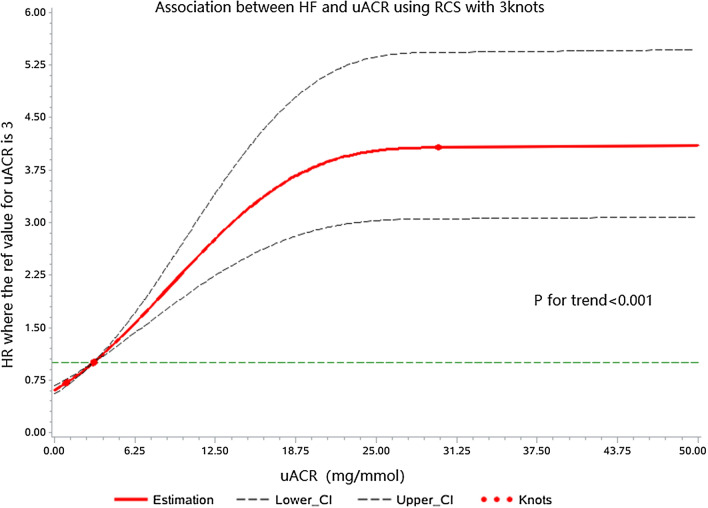
Adjusted relative hazard of heart failure by the continuous level of urinary albumin-to-creatinine ratios (uACR). The reference point is uACR of 3 mg/mmol. The solid lines represent the hazard ratios across the spectrum of uACR. The dashed lines represent the upper and lower bounds of the 95% confidence interval. P-values reflect adjusted trends (accounting for age, sex, SBP, BMI, Total cholesterol, HbA1c, eGFR, hemoglobin, smoking, Anti-diabetic treatment, Antihypertensive treatment, CHD, and atrial fibrillation)

The overall and nonlinear associations between uACR and new-onset HF were statistically significant (*p* < 0.001). The results of the restrictive cubic spline Cox proportional hazards model indicated that the risk of HF gradually increased with an increase in uACR after adjustment for covariates (Fig. 3).

### Additional predictive value of uACR for established HF risk models

In order to investigate whether the addition of uACR to known HF risk assessment models can improve the predictivity of HF risk, it was added to model 1 as well as the WATCH-DM [18] and Williams et al. [19] study models, respectively. As shown in Table 3, the addition of uACR to the known models improved the predictivity of HF risk (*p* < 0.001).

**Table 3:** The additional predictive value of uACR for heart failure

	AUC	*P-*value	NRI	*P-*value	IDI	*P-*value
Model 2	0.680 (0.665, 0.715)	–	Ref.	–	Ref.	–
Model 2 + ln(uACR)	0.783 (0.754, 0.813)	< 0.001	0.330 (0.237, 0.404)	< 0.001	0.034 (0.022, 0.052)	< 0.001
Model 2 + uACR categories	0.768 (0.737, 0.799)	< 0.001	0.362 (0.296, 0.424)	< 0.001	0.024 (0.014, 0.039)	< 0.001
Model 4	0.744 (0.710, 0.776)	–	Ref.	–	Ref.	–
Model 4 + ln(uACR)	0.802 (0.773, 0.832)	< 0.001	0.291 (0.200, 0.369)	< 0.001	0.037 (0.021, 0.061)	< 0.001
Model 4 + uACR categories	0.793 (0.763, 0.823)	< 0.001	0.284 (0.209, 0.358)	< 0.001	0.025 (0.012, 0.042)	< 0.001
Model 5	0.755 (0.721, 0.788)	–	Ref.	–	Ref.	–
Model 5 + ln(uACR)	0.807 (0.777, 0.837)	< 0.001	0.285 (0.182, 0.347)	< 0.001	0.039 (0.021, 0.060)	< 0.001
Model 5 + uACR categories	0.798 (0.767, 0.830)	< 0.001	0.303 (0.231, 0.375)	< 0.001	0.025 (0.012, 0.042)	< 0.001

Model 2: age, sex; Model 4 (WATCH-DM risk score): age, BMI, SBP, DBP, FPG, serum creatinine, HDL cholesterol, CHD; Model 5(Williams et al. study model): age, SBP, CHD, Atrial fibrillation, HbA1c, Albumin, BUN, eGFR, smoking;

*uACR* urine albumin-to-creatinine ratio, *AUC* the area under the receiver operating characteristic curve, *NRI* net reclassification improvement, *IDI* integrated discrimination improvement

### Multivariate Cox regression subgroup analysis of uACR effect on new-onset HF

Among the factors influencing HF, uACR was not significantly interactive with sex, SBP, and HbA1c (*p* > 0.05) but was interactive with eGFR (*p* < 0.05). Multivariate Cox regression analysis was performed in gender, renal function (assessing eGFR levels), SBP, and HbA1c subgroups, respectively. Our results revealed that the incidence and risk of HF in each population increased with an increase in uACR and were consistent in the overall population (Table 4).

**Table 4:** Hazard ratios (HR) and 95% Confidence intervals of uACR for heart failure (subgroup analysis)

	No.	Incident heart failure (%)	Incidence rate (/1000 person-years)	< 3 mg/mmol	3–30 mg/mmol	≥ 30 mg/mmol	Interaction *p* value
Gender							0.054
Male	6754	146	4.45	Ref.	2.50 (1.65, 3.77)	7.79 (4.91, 12.36)	
Female	2533	70	5.60	Ref.	1.70 (1.00, 2.93)	3.54 (1.73, 7.25)	
Baseline eGFR							0.022
≥ 90	3456	61	3.92	Ref.	2.59 (1.49, 4.50)	2.21 (1.01, 6.04)	
60–90	4694	97	5.37	Ref.	1.84 (1.14, 2.96)	5.19 (2.94, 9.16)	
< 60	1137	58	14.51	Ref.	2.74 (1.21, 6.20)	15.74 (7.21, 34.36)	
Baseline SBP							0.652
SBP < 140	3582	64	4.41	Ref.	2.01 (1.18, 3.61)	2.78 (1.25, 6.19)	
140 ≤ SBP < 160	3424	75	5.59	Ref.	2.49 (1.42, 4.39)	10.37 (5.62, 19.15)	
SBP ≥ 160	2281	77	8.30	Ref.	2.14 (1.19, 3.84)	5.81 (3.06, 11.04)	
Baseline HbA1c							0.186
HbA1c < 7.0%(53.01 mmol/mol)	3857	62	4.41	Ref.	3.65 (1.99, 6.68)	13.99 (6.96, 28.16)	
HbA1c ≥ 7.0%(53.01 mmol/mol)	5430	154	6.76	Ref.	1.78 (1.21, 2.62)	4.47 (2.87, 6.97)	

Adjusted for age, sex, SBP, BMI, Total cholesterol, HbA1c, eGFR, hemoglobin, smoking, Anti-diabetic treatment, Antihypertensive treatment, CHD, and atrial fibrillation

### Sensitivity analysis

Even after adjusting for covariates and excluding all participants on anti-hypertension medication or with hypertension at baseline, uACR was still significantly associated with incident HF (all *p* < 0.001). Even after excluding individuals with CHD before baseline or during follow-up (*p* < 0.001), the relationship persisted (Additional file 1: Table S2). Also, there was no significant change in the primary result when waist circumference, but not BMI, was included in the Cox regression model (Additional file 1: Table S3).

## Discussion

Our results confirmed that elevated uACR is an independent risk factor for new-onset HF in patients with T2D, irrespective of sex, renal function strata, SBP strata, HbA1c strata, and the presence of hypertension or CAD. Furthermore, uACR was also associated with the risk of HF in a dose–response manner. Additionally, our results proved that adding uACR to established HF risk models can improve their predictive ability for HF risk.

Our important finding revealed that elevated uACR is a significant risk factor for HF in type 2 diabetic patients; when uACR is mildly elevated (3–30 mg/mmol), the risk of HF increases by 2.21 fold, and when uACR ≥ 30 mg/mmol, the risk of HF increases by 6.02 fold. Previous research [20, 21] has demonstrated that the risk of HF in diabetic patients increased with the increase in urinary albumin excretion at 24 h. Current guidelines recommend measuring uACR in spot urine samples, which has a comparable diagnostic value to the urinary protein quantification at 24 h [22, 23]. To our knowledge, this is the first study to demonstrate that elevated uACR is an independent risk factor for new-onset HF in patients with T2D. Although few previous studies had similar results, the ARIC [11] and SPRINT [24] studies demonstrated a 2.49–2.75 fold and 3.47–4.76 fold higher risk of HF in people with microalbuminuria and macroalbuminuria than in those without albuminuria in the general population, respectively.

We not only verified uACR elevation as an independent risk factor for HF in patients with T2D but also found a dose–response relationship between uACR and HF risk. In type 2 diabetic patients, the risk of HF increased significantly with an increase in uACR even below the clinically defined microalbuminuria threshold (3 mg/mmol), while the HF risk increased relatively slowly when uACR elevated to about 25 mg/mmol. ARIC study [11] also demonstrated that the HF risk increased when uACR was at a high normal value (about 1–3 mg/mmol) in the general population, while the HF risk increased relatively slowly after uACR exceeded about 30 mg/mmol.

Although eGFR and uACR are both sensitive markers for renal function and independent risk factors for HF [25], our results revealed that uACR and eGFR were two interactive factors affecting HF. However, the subgroup analysis by eGFR category showed that increasing uACR increased the risk of HF more significantly as eGFR decreased; the risk of HF increased by 15.74 fold in people with uACR ≥ 30 mg/mmol and eGFR < 60 mL/min/1.73 m^2^. These risk values were consistent with that of the general population, but they were significantly higher in diabetic patients than in the general population [11, 24]. Additionally, the elevation of uACR had the strongest increasing effect on HF risk in people with a baseline SBP of 140–160 mmHg or HbA1c < 7% (53.01 mmol/mol), which might be because these people received more intensive antihypertensive or hypoglycemic therapies in clinical practice.

In recent years, many epidemiological surveys and clinical studies on HF risk factors have shown that in addition to traditional risk factors such as age, CHD, hypertension, hyperglycemia, various risk factors closely related to the pathogenesis of HF need to be further studied and confirmed. In this study, we added uACR to the WATCH-DM risk score [18] from the ACCORD test and Williams et al. [19] HF risk prediction model in diabetic patients. Our results confirmed that the addition of uACR in validated models could improve the prediction efficacy of HF risk in patients with T2D, which was consistent with the findings of Nowak et al. [14] using the ARIC HF prediction model in the general population. Our results suggest that uACR can provide a predictive value beyond the traditional risk factors for HF in patients with T2D, so uACR should be monitored regularly in the early stages of diabetes.

The possible mechanisms underlying the high risk of HF in diabetic patients include both macroangiopathy and microangiopathy. Firstly, diabetes mellitus acts as a risk factor for coronary atherosclerosis [26] and can lead to HF through CHD. Secondly, the myocardial damage caused by diabetes mellitus mainly involves small and medium-sized microvessels and plays a vital role in vascular endothelial function, including endothelial proliferation, subendothelial fibrosis; thus, decreasing the reactivity of myocardial small vessels to vasoactive substances and causing coronary small vessel hypoperfusion [27]. We found that the HF risk increased more significantly with increased uACR after excluding patients with baseline CHD and new-onset CHD during follow-up, with a 2.40 (1.64–3.50) fold and 6.61 (4.28–10.20) fold higher risk of HF in people with microalbuminuria and macroalbuminuria than in people with normal uACR. These results indicate that the high risk of HF in diabetic patients cannot be entirely explained by coronary atherosclerosis, and microcirculatory disturbance might be present in their myocardium. Thus, an increase in uACR may reflect cardiac microangiopathy of the myocardium in the absence of coronary artery disease, subsequent pathological left ventricular hypertrophy, and myocardial remodeling [28–30].

Several studies have confirmed a significant clustering between elevated uACR and traditional risk factors for HF, which include insulin resistance [31], inflammatory response [32], and renin-angiotensin- aldosterone system (RAAS) activation [33].

Considering the higher mortality rate in patients with T2D than in the general population, impending death may generate a competing risk, so we established a competing risk model for mortality in the overall population and obtained consistent and reliable results with the main model. However, there are no studies on the effect of uACR on new-onset HF analyzed by a competing risk model for mortality to date.

Our research had some limitations. At first, all HF events were hospitalized and relied on hospital diagnostic coding. This outcome may have excluded HF patients who were never admitted to the hospital. While we had information on HF hospitalizations, there was no echocardiographic data, we could not distinguish HF with preserved ejection fraction from HF with reduced ejection fraction. A previous clinical trial found that proteinuria increases the hospitalization rate of HF patients [9]^.^ Our findings may be useful in the prediction, evaluation, and treatment decision-making of high-risk HF populations with clinically detected proteinuria. Second, the proportion of each type of antihypertensive drug counted in this study was low, to avoid the impact of this deficiency on our results, we adjusted for antihypertensive drugs (yes or no) in the regression model. While, after adjusting for the use of angiotensin-converting enzyme inhibitors (ACEI) and/or angiotensin II receptor blockers (ARB), β-blockers, diuretics, and the exclusion of individuals taking antihypertensive medications, sensitivity analyses produced results that were consistent with our primary analyses. Furthermore, The proportion of participants receiving anti-diabetic treatment was low in this study, which may affect the endpoints of the study, particularly HF. Finally, because the study participants were mostly male Kailuan Group employees, the extrapolation of results may be limited. However, the results in the male and female populations were both consistent with those in the overall population after gender subgrouping.

## Conclusion

This study confirmed the independent predictive value of elevated uACR in T2D patients for an increased risk of HF, which can help to explain the high risk of HF in T2D patients and provide a useful reference for screening high-risk HF populations and assessing HF risk in T2D patients.

## Supplementary Information

**Additional file 1: Table S1. **Cox proportional-hazards model (death competitive risk model) affecting HF. **Table S2. **Hazard ratios (HR) and 95% Confidence intervals of uACR for heart failure (sensitivity analysis). **Table S3. **Hazard ratios (HR) and 95% Confidence intervals of uACR for heart failure (sensitivity analysis).

## Data Availability

The datasets used and/or analyzed during the current study are available from the corresponding author on reasonable request.

## Abbreviations

uACR: Urine albumin-to-urine creatinine ratio
HF: Heart failure
T2D: Type 2 diabetes
AUC: Area under the receiver operating characteristic curve
NRI: Net reclassification improvement
IDI: Integrated discrimination improvement
SD: Standard deviation
CHD: Coronary heart disease
BMI: Body mass index
eGFR: Estimated glomerular filtration rate
CKD-EPI: Chronic Kidney Disease Epidemiology Collaboration
ADA: American Diabetes Association
FBG: Fasting blood glucose
HbA1c: Hemoglobin A1c
SBP: Systolic blood pressure
DBP: Diastolic blood pressure
LDL: Low-density lipoprotein
HDL: High-density lipoprotein
BUN: Blood urea nitrogen
hs-CRP: High sensitivity C-reactive protein
ACEI: Angiotensin-converting enzyme inhibitor
ARB: Angiotensin receptor blocker
WATCH-DM: Weight [BMI], Age, hypertension, Creatinine, HDL-C, Diabetes control [fasting plasma glucose], QRS Duration, MI, and CABG
