# Supplementary material for: Elevated urine albumin-to-creatinine ratio increases the risk of new-onset heart failure in patients with type 2 diabetes
Source: Cardiovasc Diabetol. 2023 Mar 25;22:70. doi: 10.1186/s12933-023-01796-6 (PMC10040119; doi:10.1186/s12933-023-01796-6)
Supplement: Supplementary file 1 — Additional file 1: Table S1. Cox proportional-hazards model (death competitive risk model) affecting HF. Table S2. Hazard ratios (HR) and 95% Confidence intervals of uACR for heart failure (sensitivity analysis). Table S3. Hazard ratios (HR) and 95% Confidence intervals of uACR for heart failure (sensitivity analysis). [file 12933_2023_1796_MOESM1_ESM.docx]

**Additional file Material**

**Table S1.** Cox proportional-hazards model (death competitive risk model) affecting HF.

|  | No. | Unadjusted | Model 2 | Model 3 |
| --- | --- | --- | --- | --- |
| <3mg/mmol | 6120 | ref | ref | ref |
| 3-30 mg/mmol | 2611 | 1.20(0.87, 1.64) | 1.20(0.88, 1.65) | 1.20(0.86, 1.68) |
| ≥30 mg/mmol | 556 | 2.06(1.46, 2.90) | 2.07(1.47, 2.92) | 2.01(1.34, 3.02) |
| Per +1 sd ln(uACR) | 9287 | 1.30(1.18, 1.44) | 1.31(1.18, 1.45) | 1.33(1.18, 1.51) |

Model1: unadjusted; Model 2: adjusted age and sex; Model 3: adjusted age, sex, SBP, BMI, Total cholesterol, HbA1c, eGFR, hemoglobin, smoking, Anti-diabetic treatment, Anti-hypertensive treatment, CHD and atrial fibrillation.

**Table S2.** Hazard ratios (HR) and 95% Confidence intervals of uACR for heart failure (sensitivity analysis)

| uACR category | No hypertension  (n=4015) | | | No CHD  (n=8794) | | No hypertension or CHD  (n=3802) | |
| --- | --- | --- | --- | --- | --- | --- | --- |
|  | HR(95%CI) | *P* value | | HR(95%CI) | *P* value | HR(95%CI) | *P* value |
| <3 mg/mmol | 1 |  | | 1 |  | 1 |  |
| 3-30mg/mmol | 1.98(1.18, 3.32) | 0.009 | | 2.36(1.64, 3.38) | <0.001 | 2.60(1.45, 4.67) | 0.001 |
| ≥30 mg/mmol | 5.26(2.82, 9.83) | <0.001 | | 6.15(4.04, 9.36) | <0.001 | 7.08(3.56, 14.09) | <0.001 |
|  |  |  | |  |  |  |  |
| uACR categories | Without CHD before baseline or during follow-up  (n=8703) | | | adjusted types of antihypertensive drugs ^*^  （n=9287） | | Excluding patients under treatment of anti-hypertensive drugs  (n=5388) | |
|  | HR(95%CI) | | *P* value | HR(95%CI) | *P* value | HR(95%CI) | *P* value |
| <3 mg/mmol | 1 |  | | 1 |  | 1 |  |
| 3-30mg/mmol | 2.40(1.64, 3.50) | <0.001 | | 2.19(1.58, 3.04) | <0.001 | 2.31(1.44, 3.69) | <0.001 |
| ≥30 mg/mmol | 6.61(4.28,10.20) | <0.001 | | 5.85(3.99, 8.59) | <0.001 | 6.58(3.73, 11.60) | <0.001 |

Note: adjusted age, sex, SBP, BMI, total cholesterol, HbA1c, eGFR, hemoglobin, smoking, anti-diabetic treatment, anti-hypertensive treatment, CHD and atrial fibrillation. * adjusted ACEI/ARB, beta-blocker and diuretic.

Abbreviation: uACR: urinary albumin-to-creatinine ratios;

**Table S3.** Hazard ratios (HR) and 95% Confidence intervals of uACR for heart failure (sensitivity analysis)

| uACR Category | No. | Model 3 |
| --- | --- | --- |
| <3 mg/mmol | 6120 | 1 |
| 3–30 mg/mmol | 2611 | 2.20(1.58, 3.05) |
| ≥30 mg/mmol | 556 | 5.87(4.01, 8.61) |

Note: adjusted age, sex, SBP, waist circumference, total cholesterol, HbA1c, eGFR, hemoglobin, smoking, anti-diabetic treatment, anti-hypertensive treatment, CHD and atrial fibrillation.
